# Short-Term Alternate Feeding between Terrestrially Sourced Oil- and Fish Oil-Based Diets Modulates the Intestinal Microecology of Juvenile Turbot

**DOI:** 10.3390/biology12050650

**Published:** 2023-04-26

**Authors:** Xiuhua Ma, Yaoyao Kong, Houguo Xu, Qingzhu Bi, Mengqing Liang, Kangsen Mai, Yanjiao Zhang

**Affiliations:** 1 Key Laboratory of Aquaculture Nutrition and Feed (Ministry of Agriculture and Rural Affairs) & Laboratory of Mariculture (Ministry of Education), Ocean University of China, Qingdao 266003, China; 2 Yellow Sea Fisheries Research Institute, Chinese Academy of Fishery Sciences, Qingdao 266071, China

**Keywords:** dietary lipid source, soybean oil, beef tallow, gastro-intestinal tract, intestinal health, *Scophthalmus maximus*

## Abstract

**Simple Summary:**

There have been reports on alternate feeding between diets based on different lipid sources, but how the intestinal microbiota of fish responds to alternate feeding strategies and its relevant roles in the host’s health have not been reported. The present study provides novel results in this area. The main objective of the study was to assess how the intestinal microbiota of fish responds to alternate feeding between diets based on different lipid sources. Foremost, the juvenile turbot accepted alternate feeding between diets based on different lipid sources with no negative effects on survival or growth performance. Additionally, novel results were observed regarding the different bacterial compositions, microbial co-occurrence networks, and functional predictions among the different alternating feeding strategies. The results of this study indicate that a more comprehensive evaluation should be conducted from the perspective of intestinal microorganisms when a new feeding strategy is applied in aquaculture practices. These results will contribute to the nutritional regulation of aquatic animals from the perspective of microorganisms.

**Abstract:**

A nine-week feeding trial was conducted to investigate changes in the intestinal microbiota of turbot in response to alternate feeding between terrestrially sourced oil (TSO)- and fish oil (FO)-based diets. The following three feeding strategies were designed: (1) continuous feeding with the FO-based diet (FO group); (2) weekly alternate feeding between soybean oil (SO)- and FO-based diets (SO/FO group); and (3) weekly alternate feeding between beef tallow (BT)- and FO-based diets (BT/FO group). An intestinal bacterial community analysis showed that alternate feeding reshaped the intestinal microbial composition. Higher species richness and diversity of the intestinal microbiota were observed in the alternate-feeding groups. A PCoA analysis showed that the samples clustered separately according to the feeding strategy, and among the three groups, the SO/FO group clustered relatively closer to the BT/FO group. The alternate feeding significantly decreased the abundance of *Mycoplasma* and selectively enriched specific microorganisms, including short-chain fatty acid (SCFA)-producing bacteria, digestive bacteria (*Corynebacterium* and *Sphingomonas*), and several potential pathogens (*Desulfovibrio* and *Mycobacterium*). Alternate feeding may maintain the intestinal microbiota balance by improving the connectivity of the ecological network and increasing the competitive interactions within the ecological network. The alternate feeding significantly upregulated the KEGG pathways of fatty acid and lipid metabolism, glycan biosynthesis, and amino acid metabolism in the intestinal microbiota. Meanwhile, the upregulation of the KEGG pathway of lipopolysaccharide biosynthesis indicates a potential risk for intestinal health. In conclusion, short-term alternate feeding between dietary lipid sources reshapes the intestinal microecology of the juvenile turbot, possibly resulting in both positive and negative effects.

## 1. Introduction

Fish oil (FO) is the most important lipid source in aqua feeds [1]. However, due to supply shortages and the rising costs of FO, terrestrially sourced oils (TSOs) have been widely used as alternative lipid sources in fish feeds [2]. Soybean oil (SO) and beef tallow (BT) are important TSOs. However, an FO replacement with high levels of SO or BT usually results in decreased growth performance, excessive hepatic lipid deposition, reduced n-3 long-chain polyunsaturated fatty acid (LC-PUFA) contents, and increased inflammatory responses [3,4,5,6]. Feeding strategies are being modulated to spare FO in fish feeds. Alternate feeding between FO- and TSO-based diets is receiving increasing attention. Francis et al. reported that the retention and deposition of EPA and DHA in Murray cod (*Maccullochella peelii*) were more efficient when canola oil- and FO-based diets were regularly alternated in a series of weekly cycles [7]. Eroldoğan et al. also investigated the same feeding strategy in gilthead sea bream (*Sparus aurata*) and reported that the growth performance and the EPA and DHA contents were elevated when gilthead sea bream were subjected to a daily alternation of canola oil- and fish oil-based diets [8]. Similarly, our previous studies have confirmed that alternate feeding weakened the negative effects of TSO on the growth performance and fillet quality of turbot [9]. However, how the intestinal microbiota of fish responds to alternate feeding strategies and its relevance to the host’s health have not been reported.

Intestinal microbiota play a vital role in intestinal health and nutrient metabolism [10,11,12]. Complex and relatively stable microbe–microbe and host–microbe relationships exist in animal gastrointestinal microecosystems [13,14,15]. The composition of the intestinal microbiota of marine fish can be affected by many factors. These factors include host ones, such as genotype, physiological status, and nutritional level, as well as environmental ones, such as diet, water temperature, and water salinity [16,17,18]. It has been reported that dietary lipids can alter the intestinal microbiota of marine fish [19,20]. In the present study, we adopted a feeding strategy for juvenile turbot where TSO- and FO-based diets were alternated weekly. The present study was aimed at assessing how the intestinal microbiota of fish responds to alternate feeding between TSO- and FO-based diets. The microbe–microbe and host–microbe interactions under this feeding strategy were also investigated. These results will contribute to the nutritional regulation of aquatic animals from the perspective of microorganisms.

## 2. Materials and Methods

### 2.1. Experimental Diets

The experimental diets based on FO, SO, and BT, respectively, were formulated and contained approximately 12% crude lipid and 50% crude protein (Table 1; the fatty acid composition is shown in Table 2). The FO-based diet was used as the control diet. In the other two experimental diets, FO was completely replaced by SO or BT. The diets were prepared as previously described [21].

### 2.2. Fish Husbandry

**Figure 1:**
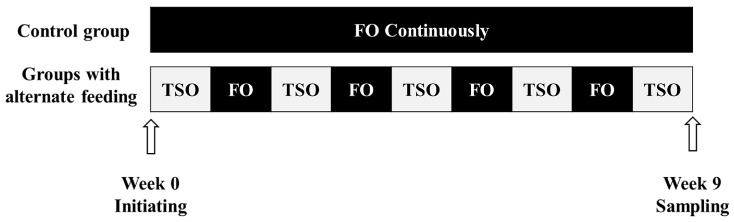
Flowchart of the feeding trial. Sampling for the present study was conducted at the end of week 9.

The healthy juvenile turbot used in this experiment were obtained from Kehe Ocean Co., Ltd. (Weihai, Shandong Province, China). Before the feeding experiment, the fish were acclimated to the experimental conditions for 7 days, during which they were fed a low-lipid commercial diet. Then, the fish with similar sizes (an initial mean weight of 26 g) were randomly assigned to nine polyethylene tanks (500 L; 35 fish per tank). All of the fish were cultured in a flow-through seawater system (indoor). Three replica tanks were randomly assigned to each feeding strategy. The seawater was pumped from the deep well, aerated, and then pumped into the fish rearing system. The feeding schedules were as follows: (1) the FO group (control) was fed with the FO-based diet continuously; (2) the SO/FO group was fed with weekly alternations of the SO- and FO-based diets; and (3) the BT/FO group was fed with weekly alternations of the BT- and FO-based diets. The 9-week feeding trial started and ended with the TSO-based diets (Figure 1). Following the principle of apparent satiation feeding, the fish were fed twice daily at 7:30 and 17:30. A third of the rearing water was changed after 2 h of each feeding. During the experiment, the conditions were as follows: the water temperature was 13.2–15.2 °C; the dissolved oxygen was 6–8 mg/L; the salinity was 27–29‰; and the pH was 7.5–8.0.

### 2.3. Sample Collection

At the end of week 9 and after 24 h of starvation, the fish were weighted. Considering that our study aimed for an intestinal autochthonous microbial composition, as described in our previous study, the 24 h hunger period ensures intestinal emptying in the turbot [9]. Two fish per tank (six fish per group) were randomly selected for the intestinal microbiota analysis. In brief, the exterior of the fish was wiped with 70% alcohol. After that, the hindgut was dissected with sterile anatomical tools near an alcohol burner and transferred into 2 mL sterile tubes (Corning, Corning, NY, USA), frozen in liquid nitrogen, and stored at −80 °C before use. All animal care and handling protocols were approved by the Animal Care and Use Committee of the Yellow Sea Fisheries Research Institute (recorded case number: IACUC202003154258).

### 2.4. Intestinal Microbiota DNA Extraction, 16S rRNA Sequencing, and Data Analysis

The bacterial DNA was extracted using the QIAamp^®^ Fast DNA Stool Mini Kit (Qiagen, Hilden, Germany), according to the manufacturer’s instructions. Detailed information on DNA extraction and sequencing can be found in a previously published article [9]. To evaluate the diversity of the host microorganisms and the differences among the microbial communities, alpha and beta diversity analyses were performed. The dissimilarity among the microbial communities was evaluated with the unweighted UniFrac distance. A MetaStat analysis identified genera with different abundances between the groups. In addition, a co-occurrence network analysis was assessed by calculating the relative abundance matrix based on the genus level using the psych package in R, version 3.6.0. To compare the co-occurrence patterns of the bacteria, three networks were generated based on the feeding strategies. The Spearman’s rank correlation coefficient (*r* > 0.6 at *p* < 0.05) was utilized to calculate the correlation. Based on the calculated correlation, the networks were constructed with the nodes representing the bacterial genera and the edges representing the significant correlation (positive or negative interactions) between the nodes. Furthermore, the number of nodes and edges, graph density, modularity, average path length, and average degree were calculated for each network. The correlation matrix was visualized using Gephi 0.9.2.

### 2.5. Functional Predictions of Intestinal Microbiota

The Phylogenetic Investigation of Communities by Reconstruction of Unobserved States (PICRUSt 2.3.0-b) was used to predict the functional profiles of the microbial communities under the three feeding strategies. Then, by standardizing the copy number of the data with a new OTU table, the relative Kyoto Encyclopedia of Genes and Genomes (KEGG) pathway abundance was obtained. The output files of the PICRUSt used STAMP (Statistical Analysis of Metagenomic Profiles) for the statistical analysis and visualization. Welch’s two-sided *t*-test was used to predict the significant differences in the metagenomic pathway, and Benjamini–Hochberg FDR was used to eliminate the KEGG false-positive pathway generated by the multiple comparisons.

### 2.6. Calculation and Statistical Methods

Survival = final number of fish/initial number of fish × 100

Weight gain = (final weight − initial weight)/initial weight × 100

Feed efficiency ratio = (final weight − initial weight)/total dry feed × 100

Viscerosomatic index (VSI) = wet viscera weight/fish body weight × 100

Hepatosomatic index (HSI) = wet liver weight/fish body weight × 100

In addition to the microbiota sequencing data, other statistical analyses were performed using SPSS 26.0. The data were subjected to a one-way analysis of variance (ANOVA), followed by a Tukey’s test. The results were presented as means ± standard errors. A *p* value of *<* 0.05 in the statistical tests was considered significant.

## 3. Results

### 3.1. Growth Performance and Somatic Parameters

At the end of the feeding trial, the alternate feeding between the TSO- and FO-based diets did not induce alterations in the growth performance or somatic parameters of the juvenile turbot. As shown in Table 3, there were no significant differences in the final weight, survival rate, weight gain, feed efficiency ratio, VSI, or HSI among the groups (*p* > 0.05).

### 3.2. OTU Taxonomic Statistics and Venn Diagrams

The statistical results of the OTU classifications showed that a total of 8188 OTU sequences were generated with ≥97% sequence similarity. A total of 1071 genera, 485 families, 317 orders, 144 classes, and 66 phyla were obtained. The rarefaction curves approached the saturation plateau, indicating that all samples were sequenced completely (Figure S1). It can be seen from the Venn diagram that 3221 OTUs were shared by each group. The OTU numbers unique to the FO, SO/FO, and BT/FO groups were 639, 1017, and 1386, respectively (Figure S2).

### 3.3. Effects of Feed Alteration Strategies on Intestinal Microbiome Structure

**Figure 2:**
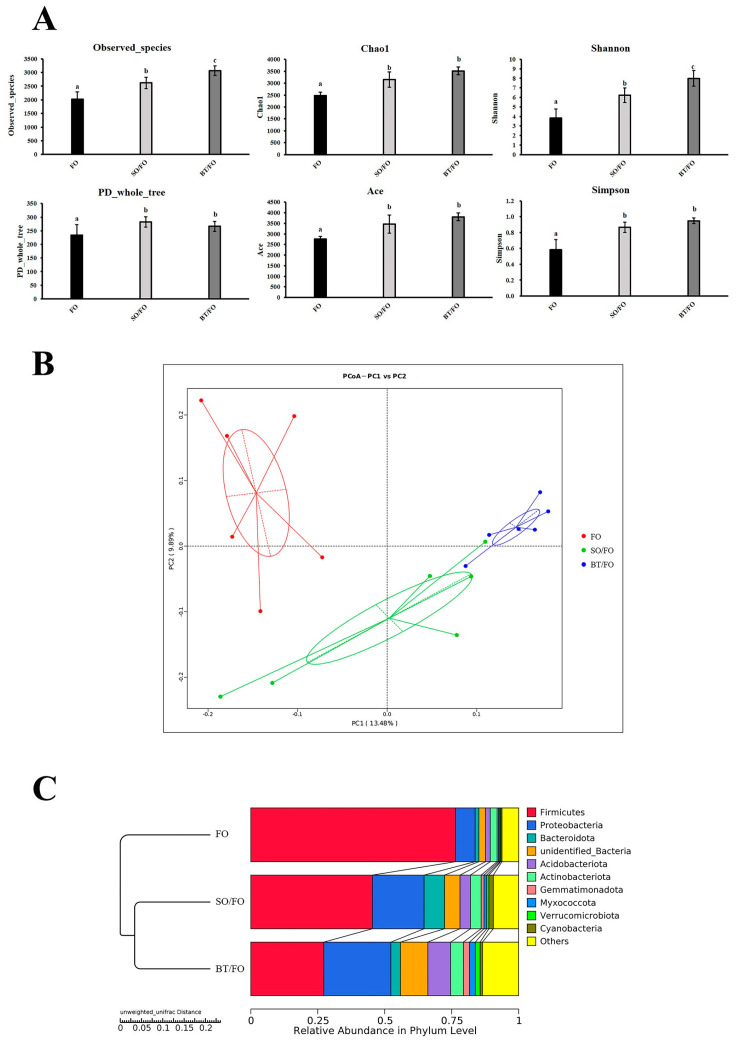
Alpha and beta diversity indices of the intestinal microbiota of turbot under different feeding schedules (six replicates per group). (**A**) Alpha diversity index of the intestinal microbiota of the experimental turbot. The results were expressed as means ± S.E., and the different letters (a, b, and c) above the bars indicate significant differences (*p* < 0.05). (**B**,**C**) Beta diversity of the intestinal microbiota of the turbot under different feeding schedules. The principal coordinate analyses (PCoAs) using PC1 versus PC2 axes and the UPGMA clustering trees of the samples were all based on the unweighted UniFrac distance. The line and circle indicate the cluster of samples calculated based on the unweighted UniFrac distance.

The alpha diversity indices, including the Chao1 index, ACE index, Shannon diversity, and Simpson diversity, were significantly higher in the intestinal microbiota of the fish in the SO/FO and BT/FO groups (Figure 2A). The principal coordinate analysis (PCoA) showed that the samples clustered separately based on the diets (Figure 2B). The intestinal bacterial compositions of the SO/FO and BT/FO groups were distinctly separated from the FO group, but the SO/FO group clustered relatively closer to the BT/FO group (Figure 2B). Moreover, a similar result could be observed in the unweighted pair-group method with arithmetic mean (UPGMA) analyses (Figure 2C).

### 3.4. Effects of Alterative Feeding on Intestinal Microbiome Composition

**Figure 3:**
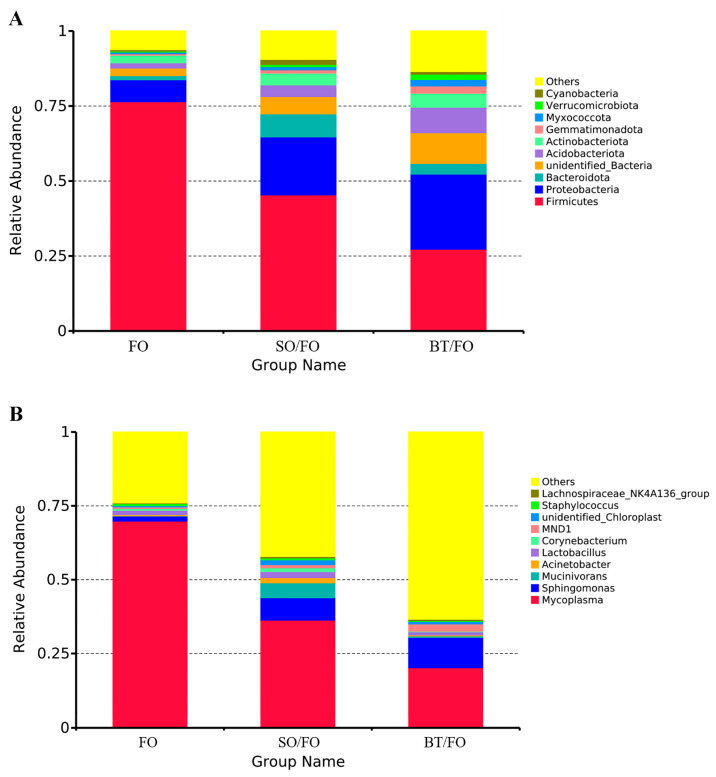
Top ten most abundant (based on relative abundance) bacteria of the intestinal microbiota at the phylum (**A**,**B**) genus levels (six replicates per group).

At the phylum level, the top ten dominant bacterial phyla in the three groups included Firmicutes, Proteobacteria, Bacteroidota, Acidobacteriota, Actinobacteriota, Gemmatimonadota, Myxococcota, Verrucomicrobiota, Cyanobacteria, and unidentified_Bacteria (Figure 3A). At the genus level, *Mycoplasma*, *Sphingomonas*, *Mucinivorans*, *Acinetobacter*, *Lachnospiraceae*_NK4A136_group, *Lactobacillus*, *Corynebacterium*, *MND1*, unidentified_Chloroplast, and *Staphylococcus* comprised the top ten abundant intestinal genera of the intestinal bacterial community (Figure 3B).

### 3.5. Analysis of Differential Bacteria among Groups

**Figure 4:**
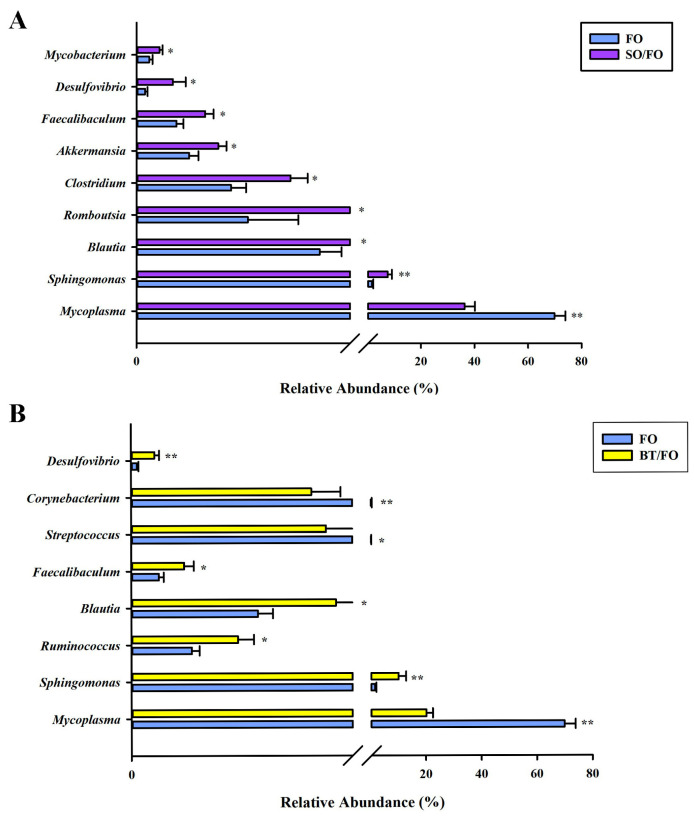
MetaStat analysis of key bacteria in the distal intestinal mucosa of the experimental turbot (six replicates per group). (**A**) MetaStat analysis of the bacterial communities between the FO and SO/FO groups. (**B**) MetaStat analysis of the bacterial communities between the FO and BT/FO groups. * means significant differences between the FO group and the SO/FO or BT/FO groups, evaluated by a *t*-test (* *p* < 0.05; ** *p* < 0.01).

Additional MetaStat analyses at the genus level showed significant changes in the intestinal microbiome among the different groups. Compared to the control group, the SO/FO group significantly (*p* < 0.05) increased the relative abundance of *Blautia*, *Clostridium*, *Akkermansia*, *Streptococcus*, *Romboutsia*, *Faecalibaculum*, *Desulfovibrio*, and *Mycobacterium* (Figure 4A), whereas the BT/FO group significantly (*p* < 0.05) increased the relative abundance of *Blautia*, *Streptococcus*, *Ruminococcus*, *Faecalibaculum*, *Corynebacterium*, and *Desulfovibrio* (Figure 4B).

### 3.6. Microbial Co-Occurrence Network

**Figure 5:**
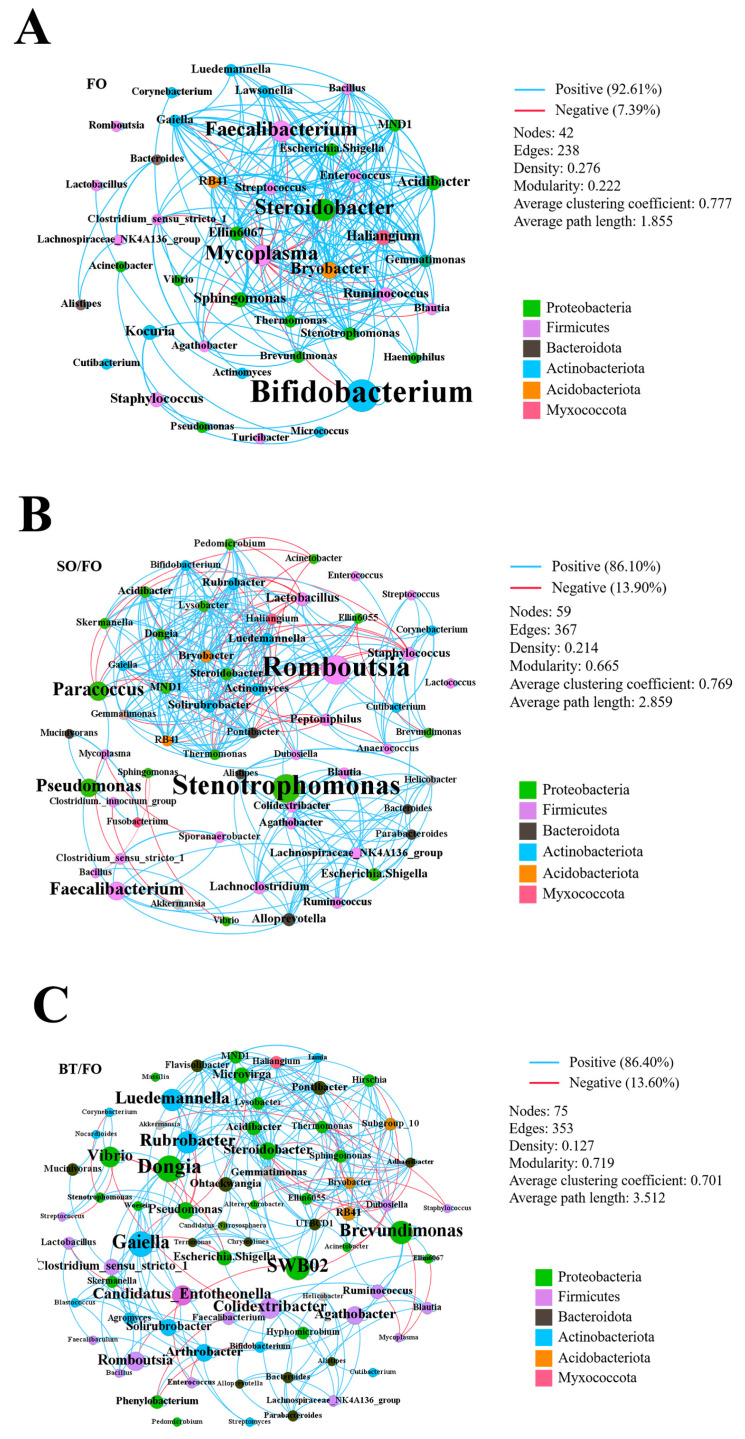
Bacterial co-occurrence network of juvenile turbot in the FO group (**A**), SO/FO group (**B**), and BT/FO group (**C**) (six replicates per group). The network diameter (of each node) corresponds to a hub value, and the weight of the color of the nodes is proportional to their relative abundance. The lines (edges and bridges between the nodes) connecting two nodes represent significant co-occurrence relationships (Spearman′s *r* > 0.60 and *p* < 0.05). The blue and red edges inside the network indicate the positive (cooperative) and negative (competitive) interactions between two bacteria, respectively. The taxa in brackets are based on annotations suggested by the Silva database.

In order to clarify whether alternate feeding is beneficial for interactions among specific bacteria and contributes to the stabilization of intestinal microbiota community structures, the co-occurrence patterns among the groups were analyzed using a network analysis. After the relative abundance of each genus was obtained, all genera with an abundance of less than 0.07% were filtered, and 42, 59, and 75 genera were obtained in the FO, SO/FO, and BT/FO groups, respectively (Figure 5). More significant co-occurrence relationships were observed in the SO/FO and BT/FO groups compared to the FO group. The dominant interactions in all three networks were positive interactions. Compared to the FO network, the competitive interactions among the intestinal bacteria were enhanced in the SO/FO and BT/FO networks. SO/FO was the most connected group, and the average degree was 12.441. Three main phyla (Firmicutes, Proteobacteria, and Bacteroidota) contributed to most networks. The modularity index and average path length were higher for the SO/FO and BT/FO groups compared with the FO group. The co-occurrence networks indicate that the complexity of the intestinal microbiota in the three groups was different.

### 3.7. Functional Predictions of Intestinal Microbiota

**Figure 6:**
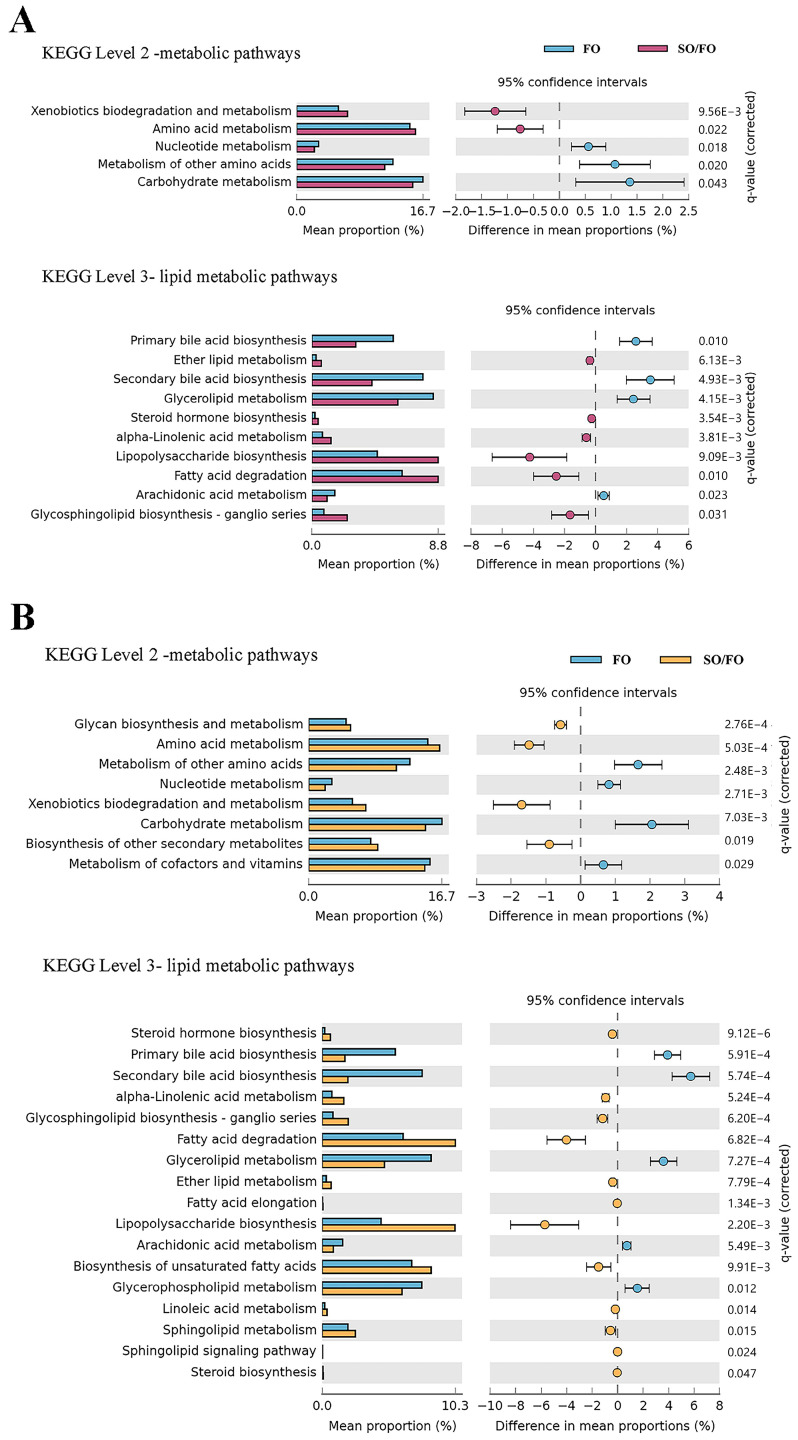
Predicted functional metagenomic pathways of the intestinal microbiome of turbot (six replicates per group) between the FO and SO/FO groups (**A**) and those between the FO and BT/FO groups (**B**). The q-value was the FDR-corrected *p*-value.

The functional potential of the intestinal microbiota of turbot under different feeding strategies at the KEGG 2 (metabolic functional gene subclasses) and KEGG 3 (specific metabolic pathways) levels was predicted using the PICRUSt software (Figure 6). Compared to the FO group, the microbial functions related to xenobiotic biodegradation and metabolism and amino acid metabolism were significantly upregulated in the SO/FO group, as well as some microbial functions involved in the biosynthesis of other secondary metabolites, amino acid metabolism, glycan biosynthesis and metabolism, and xenobiotic biodegradation and metabolism were significantly upregulated in the BT/FO group. In addition, in the lipid metabolic pathways, the abundance of pathways related to lipopolysaccharide biosynthesis, ether lipid metabolism, fatty acid degradation, steroid hormone biosynthesis, and alpha-linolenic acid (ALA) metabolism was significantly higher in the SO/FO and BT/FO groups than those in the FO group. In addition, the KEGG 3 functional pathways revealed that the sphingolipid signaling pathway, fatty acid elongation, biosynthesis of unsaturated fatty acids (UFA), linoleic acid (LA) metabolism, sphingolipid metabolism, and steroid biosynthesis were enriched in the BT/FO group.

## 4. Discussion

The main objective of the study was to assess how the intestinal microbiota of fish responds to alternate feeding between TSO- and FO-based diets. Foremost, the juvenile turbot accepted the alternate feeding between TSO- and FO-based diets with no negative effects on survival or growth performance. In this study, after short-term alternate feeding between TSO- and FO-based diets, the overall intestinal microbial community of the juvenile turbot was analyzed from the aspects of structure, composition, and potential function.

Overall, the differentiation among the intestinal microbial communities among the three groups was apparent. Separate clustering of the intestinal bacterial communities was observed according to the experimental groups. Most importantly, in terms of microbial components and phylogeny, the bacteria in the SO/FO group were relatively closer to the BT/FO group. This indicates that the dynamic administration of diverse dietary lipid sources largely modified the intestinal microbial compositions. Regarding the microbial diversity, higher OTU numbers, Chao1, ACE, and Shannon and Simpson indices were observed in the SO/FO and BT/FO groups compared to the FO control group, indicating higher microbial diversity. Higher microbial diversity was generally associated with balanced intestinal microecology, stable intestinal function, and high resistance to stressful environments [22,23].

Significant differences in the microbial composition at the phylum and genus levels were also observed among the groups. The dominant phylum Firmicutes showed a significant decrease in abundance in the SO/FO and BT/FO groups, while the abundance of Bacteroidota and Proteobacteria was significantly increased in these two groups compared to the FO control group. At the genus level, in the SO/FO group, higher enrichment of the genera *Blautia*, *Clostridium*, *Akkermansia*, *Streptococcus*, *Romboutsia*, and *Faecalibaculum* was observed. They were considered to be short-chain fatty acid (SCFA)-producing bacteria. Among them, *Blautia* and *Clostridium* can produce acetate, while *Faecalibaculum* and *Romboutsia* are butyrate-producing bacteria [24,25,26]. This type of bacteria can produce SCFA through carbohydrate fermentation, providing energy and protecting the intestinal barrier [27,28,29]. In addition, some SCFA-producing bacteria belonging to the Lachnospiraceae family also regulate glucose and lipid metabolisms. *Blautia* has been shown to be involved in bile transformation, gastrointestinal function, and lipid metabolism [30,31]. *Akkermansia*, classified under the phylum Verrucomicobia, is also considered an SCFA-producing bacterium that supplies energy to intestinal goblet cells [32]. *Akkermansia* can promote mucus secretion to enhance the intestinal barrier and maintain intestinal lipid homeostasis by reducing the excessive production of chylomicrons induced by acute lipid loads [33,34]. The use of SCFAs and their salts as feed supplements can enhance the growth and health of farmed fish. This has been evidenced in Nile tilapia (*Oreochromis niloticus*), sea bream (*Sparus aurata*), yellow drum (*Nibea albiflora*, Richardson), and, most importantly, turbot [35,36,37,38].

In the BT/FO group, enrichment of SCFA-producing bacteria, including *Blautia*, *Faecalibaculum*, *Streptococcus*, *Cutibacterium*, and *Ruminococcus*, was also observed. In addition to producing secondary metabolites, such as SCFA, as described earlier, the intestinal microbiota also serves the host by contributing enzymes that improve nutrient digestion in the host [39,40]. In the present study, the relevant genera *Corynebacterium* and *Sphingomonas* were enriched in the BT/FO group. *Corynebacterium* has been reported to produce high levels of polysaccharide hydrolases and lipases [41]. *Sphingomonas* was believed to be a good producer of cellulase, protease, and amylase, providing exogenous digestive enzymes for the absorption of nutrients in the intestine [42].

Although alternate feeding exerted many favorable regulations on intestinal microecology, the increase in the abundance of pathogens, such as *Desulfovibrio* and *Mycobacterium*, by alternate feeding was also noteworthy. *Desulfovibrio*, which is a Gram-negative bacterium, produces endotoxins [43]. Hydrogen sulfide produced by these bacteria inhibits intestinal epithelial cell metabolism, impairs intestinal epithelial mucosa, and induces inflammation responses [44]. Usually, the abundance of sulfate-reducing bacteria increases after feeding on lipid-enriched or high-saturated lipid diets. This may be related to the number and chain length of some fatty acids in the feed, which remain to be further studied [45,46]. *Mycobacterium* is a Gram-positive, acid-resistant, and inactive bacterium. It causes chronic diseases in many cultured and wild fish, such as turbot, European seabass (*Dicentratrchus labrax*), and striped bass (*Morone saxatalis*) [47,48,49]. The high abundance of *Mycobacterium* in the SO/FO group may be due to the presence of phytosterols in the soybean oil, which can be used by *Mycobacterium* [50].

In spite of the enrichment of pathogens by the alternate feeding mentioned above, a lower abundance of *Mycoplasma* was observed in the intestine in two of the alternate-feeding groups, especially the BT/FO group. Some species of *Mycoplasma* have been recognized as pathogens of human and animal diseases [51]. Massive colonization of *Mycoplasma* on fish gills leads to severe damage to the gill epithelium [52,53]. Nevertheless, studies have also reported that *Mycoplasma* is part of the normal microbiota of Atlantic salmon (*Salmo salar*), which is not pathogenic in its natural host and may play an unknown role in the host’s health [54,55].

Bacteria in the intestine form a complex ecological network via their interactions with each other, and they rely on this network to keep a dynamic balance [56]. The network connections between the bacteria may be caused by the functional similarity or complementarity between the species [57]. This cooperation is unfavorable to the stability of the system due to positive feedback, whereas competition between the species can improve the stability of microecology [56]. In this study, Firmicutes and Proteobacteria have been proven to be the main components of the three types of ecological networks, occupying a key position in the network. Most importantly, the alternate feeding significantly enriched the competitive interactions. Therefore, the bacteria’s ecological networks in the SO/FO and BT/FO groups tended to be stable. The modularity, path length, and clustering coefficient were also used to evaluate the effects of alternate feeding on the ecological network of the intestinal microbial community. Modularity indicates the strength of a network divided into modules. The network with high modularity has densely connected nodes in the module [58,59]. The modularity of the SO/FO and BT/FO groups was much higher than that of the FO group, indicating that they had relatively stable intestinal microbiota homeostasis. The bacteria in the intestine rely on each other’s ecological networks to maintain a dynamic balance. The above results are based on a short-term alternate feeding trial of 9 weeks. However, according to the current information, whether a stable microbiome has been established is unknown, which may need to be further investigated by a longer feeding duration.

In addition to the profoundly altered microbial community composition, alternate feeding between dietary lipid sources also affected the nutritional metabolism of the microbiota. The results of the functional predictions of the intestinal microbiota showed that the SO/FO and BT/FO groups had more active lipid metabolism pathways, specifically the ALA metabolism. In the BT/FO group, the LA metabolism and the biosynthesis of the UFA pathways were significantly enriched. Connections between dietary fatty acid compositions and intestinal microbe functions have been observed in previous studies. The lipid metabolism (especially the biosynthesis of unsaturated and saturated fatty acids) in the intestinal microbiota was more active in Atlantic salmon when a high-lipid diet was fed for 116 days [60]. In gilthead sea bream, feeding a diet with 60% camelina oil increased the KEGG pathway of fatty acid synthesis in the intestinal microbiota [61]. Low-lipid uptake and high activation of the lipid metabolic pathways (especially LA) were also observed concurrently in wild rainbow trout (*Oncorhynchus mykiss*) populations [62]. Linoleic acid and ALA are the precursors of n-3 LC-PUFA biosynthesis. The enhancement of pathways, such as ALA metabolism, LA metabolism, and biosynthesis of UFA, in low n-3 LC-PUFA groups in the present and previous studies may compensate for the dietary n-3 LC-PUFA deficiency. Besides the fatty acid and lipid metabolisms, there was also a significant increase in the amino acid metabolism and glycan biosynthesis and metabolism in the intestinal microbiota of the SO/FO and BT/FO groups. However, the mechanisms involved warrant future studies. Beyond nutrient metabolism, there were higher levels of pathways involved in lipopolysaccharide (LPS) biosynthesis in the intestinal microbiota of the SO/FO and BT/FO groups compared to the FO group. Increased LPS biosynthesis suggests an increase in the stimulation of Gram-negative bacteria. Collectively, the increase in Gram-negative bacteria and LPS biosynthesis can induce intestinal inflammatory responses and increase the permeability of intestinal tight junctions by upregulating the expression of toll-like receptors [63]. The enrichment of LPS biosynthesis also suggests the potential risk of alternate feeding between dietary lipid sources for the intestinal health of turbot.

## 5. Conclusions

The composition and structure of the intestinal microbiota of turbot were significantly changed by weekly alternate feeding using terrestrially sourced oil- and fish oil-based diets. Alternate feeding can selectively enrich specific microorganisms, including SCFA-producing bacteria, digestive bacteria, and several potential pathogens. Alternate feeding may maintain the balance of the intestinal microbiota by improving the connectivity of the ecological network and increasing the competitive interaction within the ecological network. In addition, alternate feeding significantly affected the metabolism of the intestinal microbiota, in particular the fatty acid metabolism and LPS biosynthesis. In fish farming practices, the effects on the intestinal microbiota of fish cannot be neglected when lipid source-based alternate feeding strategies are applied.

## Figures and Tables

**Table 1:** Formulation and proximate composition of the experimental diets (% dry matter). ^1^ Mineral premix (g kg^−1^ diet): iron, 37.14; zinc, 16.43; manganese, 3.03; cuprum, 0.95; cobalt, 0.04; and iodine, 0.1, which were designed for sea fish and purchased from Qingdao Master Biotech Co., Ltd., Qingdao, China. ^2^ Vitamin premix (mg kg^−1^ diet): vitamin A, 376.2; vitamin D_3_, 4.5; vitamin E, 7600; menadione, 1200; riboflavin, 1350; vitamin B_6_, 1100; vitamin B_12_, 7.5; vitamin B_3_, 4500; biotin, 47.5; folic acid, 456; nicotinamide, 700; and inositol, 10,000, which were designed for sea fish and purchased from Qingdao Master Biotech Co., Ltd., Qingdao, China. ^3^ Containing 50% calcium propionic acid and 50% fumaric acid.

Ingredients	Fish Oil	Soybean Oil	Beef Tallow
Fish meal	40	40	40
Soy protein concentrate	5	5	5
Soybean meal	10	10	10
Wheat meal	21.98	21.98	21.98
Casein	4	4	4
Brewer’s yeast	8	8	8
Mineral premix ^1^	0.5	0.5	0.5
Vitamin premix ^2^	0.2	0.2	0.2
Monocalcium phosphate	1	1	1
L-ascorbyl-2-polyphosphate	0.2	0.2	0.2
Choline chloride	0.2	0.2	0.2
Betaine	0.3	0.3	0.3
Ethoxyquin	0.02	0.02	0.02
Mold inhibitor ^3^	0.1	0.1	0.1
Soya lecithin	1	1	1
Fish oil	7.5		
Soybean oil		7.5	
Beef tallow			7.5
Proximate composition			
Moisture	8.08	7.55	7.94
Crude protein	51.81	51.49	51.57
Crude lipid	11.56	11.87	11.70
Ash	9.50	9.76	9.46

**Table 2:** Fatty acid composition of the experimental oils and diets (% total fatty acid). ND: non-detectable.

Fatty Acid	Oil			Diet		
	Fish Oil	Soybean Oil	Beef Tallow	Fish Oil	Soybean Oil	Beef Tallow
C14:0	6.88	0.09	2.67	4.82	1.51	2.65
C16:0	20.80	11.00	36.50	18.52	13.06	24.60
C18:0	4.37	4.02	20.30	4.27	3.76	11.69
∑SFA	34.98	16.35	61.61	27.62	18.33	38.93
C16:1n-7	6.50	0.09	1.38	4.71	1.54	2.05
C18:1n-9	15.44	26.85	32.37	12.34	17.50	19.57
C20:1n-9	4.67	0.56	0.12	2.64	0.46	0.26
C22:1n-9	0.65	ND	ND	0.41	0.05	0.04
C24:1n-9	0.64	ND	ND	0.65	0.21	0.23
∑MUFA	27.90	27.49	33.87	20.75	19.76	22.15
C18:2n-6	8.04	50.50	3.86	11.84	31.37	8.37
C20:4n-6	1.06	ND	0.05	0.76	0.22	0.25
n-6∑PUFA	9.50	50.86	3.94	12.89	31.73	8.76
C18:3n-3	2.50	5.24	0.26	2.15	3.44	0.95
C20:3n-3	0.28	ND	ND	0.20	0.05	0.05
C20:5n-3	9.52	ND	ND	7.96	2.90	2.92
C22:6n-3	14.8	ND	ND	13.26	4.49	4.58
n-3∑PUFA	27.10	5.24	0.26	23.56	10.89	8.50
n-3/n-6	2.85	0.10	0.06	1.83	0.34	0.97

**Table 3:** Growth performance and somatic parameters of the experimental turbot. The values are the means of three tanks ± SEM. The mean values in the same row were not significantly different (*p* > 0.05). VSI: viscerosomatic index; HSI: hepatosomatic index.

Parameters	FO	SO/FO	BT/FO
Growth performance			
Initial weight g	25.99 ± 0.01	25.98 ± 0.03	26.01 ± 0.01
Final weight g	56.28 ± 4.31	58.92 ± 1.52	55.31 ± 9.44
Survival %	63.86 ± 5.95	56.19 ± 3.30	61.43 ± 10.10
Weight gain %	116.50 ± 16.70	126.80 ± 5.80	132.9 ± 14.10
Feed efficiency ratio	0.94 ± 0.16	0.88 ± 0.08	0.80 ± 0.08
Somatic parameters			
VSI %	5.70 ± 0.42	5.00 ± 0.08	5.72 ± 0.19
HSI %	1.35 ± 0.30	1.21 ± 0.17	1.71 ± 0.31

## Data Availability

Datasets are available on request. The raw data supporting the conclusions of this article will be made available by the authors without undue reservation.

## Supplementary Materials

The following supporting information can be downloaded at https://www.mdpi.com/article/10.3390/biology12050650/s1, Figure S1: Rarefaction curve of the intestinal microbiota of juvenile turbot, Figure S2: Venn diagram of unique and shared OTUs in the intestinal microbiota of turbot under different feeding schedules.
