# Supplementary material for: Short-Term Alternate Feeding between Terrestrially Sourced Oil- and Fish Oil-Based Diets Modulates the Intestinal Microecology of Juvenile Turbot
Source: Biology (Basel). 2023 Apr 26;12(5):650. doi: 10.3390/biology12050650 (PMC10215124; doi:10.3390/biology12050650)
Supplement: Supplementary file 1 [file biology-12-00650-s001.zip › biology-2325928-supplementary.pdf]

biology

Supplementary Materials

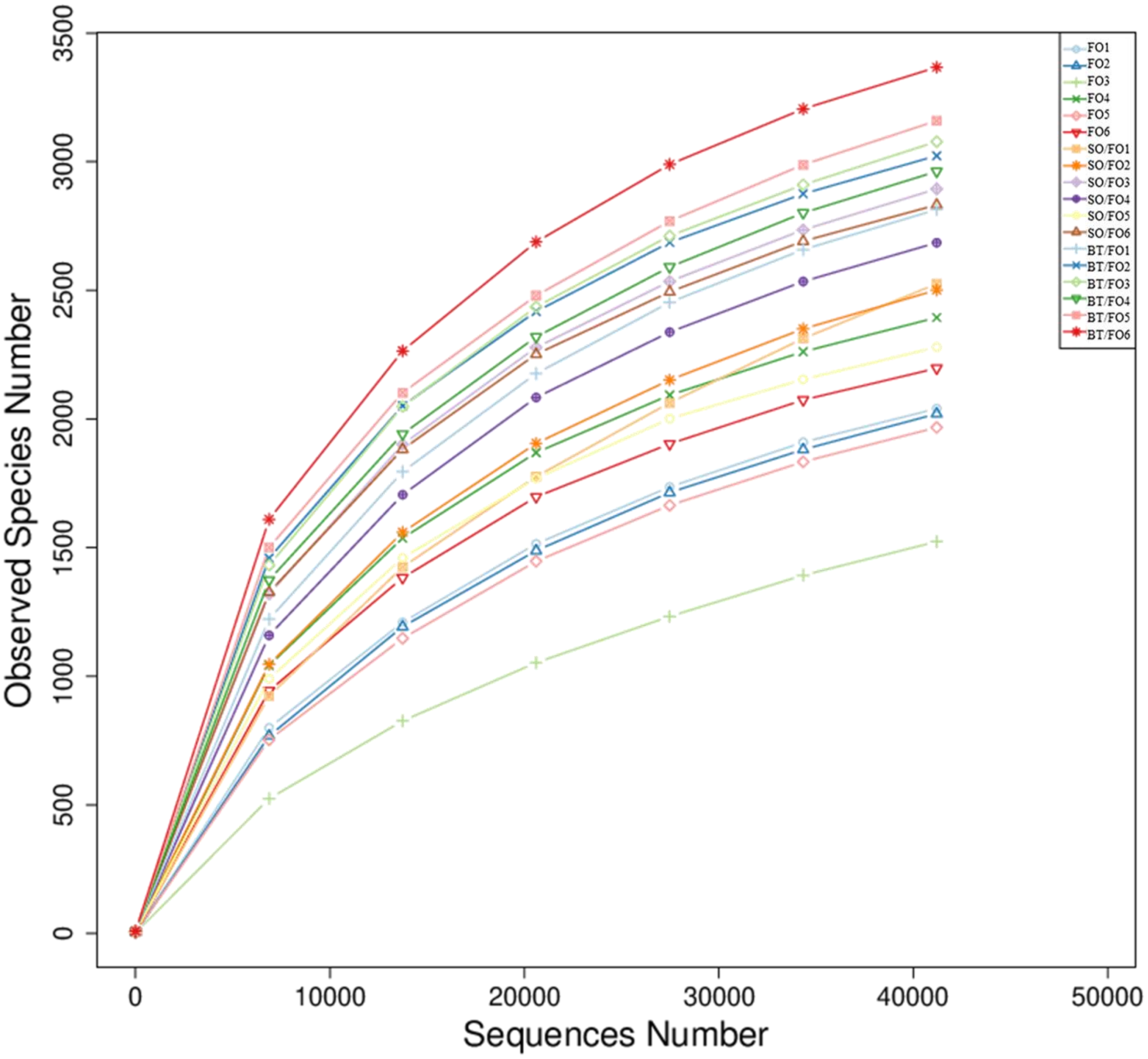

Figure S1. Rarefaction curve of intestinal microbiota of juvenile turbot.

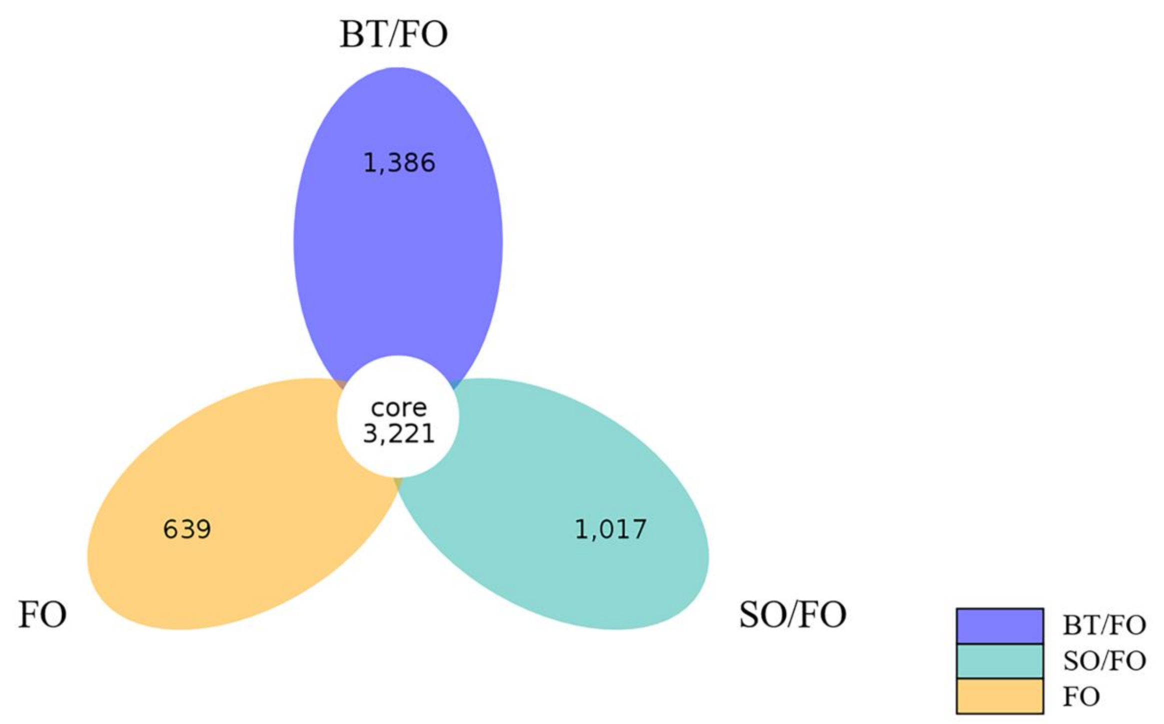

Figure S2. Venn diagram of unique and shared OTUs in the intestinal microbiota of turbot under different feeding schedules.
